# Inflammatory Myofibroblastic Bladder Tumor in a Patient with Von Recklinghausen's Syndrome

**DOI:** 10.1155/2010/523964

**Published:** 2010-08-08

**Authors:** Eleftherios Chatzidarellis, Evangelos Mazaris, Andreas Skolarikos, Demonakou Maria, Iraklis Mitsogiannis, Nafsika Mousiou, Apostolos Bisas

**Affiliations:** ^1^2nd Department of Urology Athens Medical School, Sismanoglio Hospital, Sismanogliou 1, 15126 Marousi, Athens, Greece; ^2^Department of Pathology, Sismanoglio Hospital, Sismanogliou 1, 15126 Marousi, Athens, Greece

## Abstract

Myofibroblastic tumor, also known as inflammatory pseudotumor or pseudosarcoma, is a benign tumor with mesenchymal origin. Bladder location is very uncommon. We report the case of a 58-year-old man with a history of von Recklinghausen's disease who complained for painless macroscopic hematuria 5 months after suprapubic prostatectomy. The radiograph evaluation revealed a bladder tumor, and the pathologic examination following a transurethral resection showed inflammatory myofibroblastic tumor of the bladder. The patient finally underwent a radical cystectomy due to the uncertain pathogenesis of inflammatory myofibroblastic tumor as well as the rarity of cases published on bladder tumors in Von Recklinghausen's patients.

## 1. Introduction

Inflammatory myofibroblastic tumor (IMT) or inflammatory pseudotumor (IP) of the urinary bladder is a rare benign nonepithelial lesion. This type of tumor has been described in several organs and anatomical sites including lung, head, neck, and brain. In the genitourinary system, the tumor usually originates in the bladder [1], but it has also been reported in the kidney, urethra, prostate, ureter, and rete testis. It was Roth in 1980 who first described such a lesion in the genitourinary tract [2], followed by Proppe et al. who reported similar findings in eight patients who had recently undergone a surgical procedure. The term “postoperative spindle cell nodule" (PSCN) was used to define the reactive process within three months of a surgical procedure at the site it derives [3]. Inflammatory pseudotumor (IP) on the other hand is histologically similar to PSCN, but the diagnosis is made in the absence of prior surgery [4, 5]. Regarding patient's prognosisa it is important to distinguish and differentiate this lesion from sarcoma, fact that can be difficult both clinically and histologically. The case presented here is the first of IMT in a patient with Von Recklinghausen's neurofibromatosis.

## 2. Case Presentation

**Figure 1:**
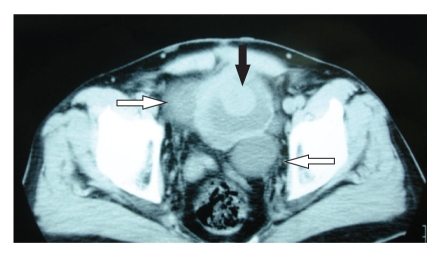
Computed tomography of the lower abdomen showing an exophytic lesion arising from the dome of the bladder (black arrow) and two large diverticulums (white arrows).

**Figure 2:**
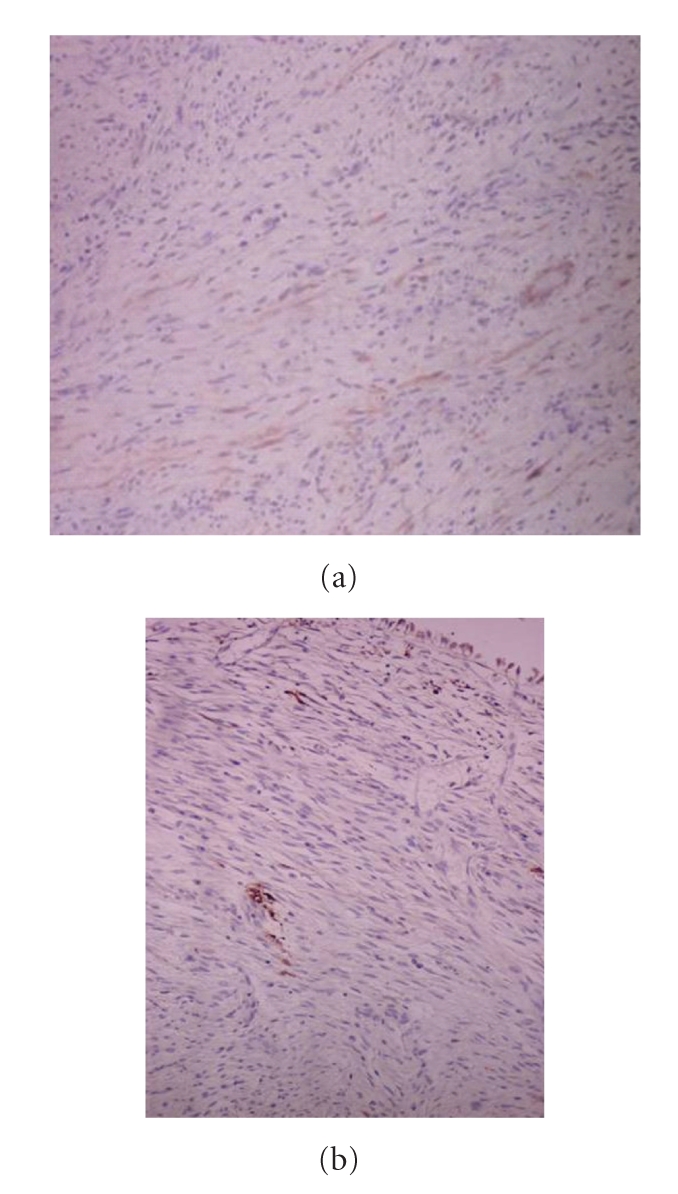
The immunohistochemical staining profile of the tumor (a) APAP/ a-SMA X 25, and (b) APAP/S-100 X 25.

 A fifty-eight-year old man was evaluated for painless macroscopic hematuria. The patient had a family history of von Recklinghausen's disease with the typical multiple cutaneous neurofibromas. Initial abdominal ultrasound revealed a tumor on the bladder dome which was confirmed by a subsequent cystoscopy. A CT scan of the abdomen showed no perivesical tissue extension, no lymph node enlargement and no hydronephrosis presence (Figure 1). Due to its uncommon endoscopically features and extension of the lesion (large base, smooth surface, and approximate size >3 cm) the patient underwent an incomplete transurethral resection of the tumor for staging purposes and to define its origin. Histopathology following tumor resection depicted a neoplasm of mesenchymal origin with immunohistochemical findings indicating the diagnosis of inflammatory myofibroblastic tumor (IMT). Vimentin was diffusely expressed in neoplastic cells and desmin, *α*-SMA, s-100, CD117, CD34 and keratins of low molecular weight were focally positive. (Figures 2(a) and 2(b)). On the other hand CD30, NSE and keratins of high molecular weight were not expressed at all. 

**Figure 3:**
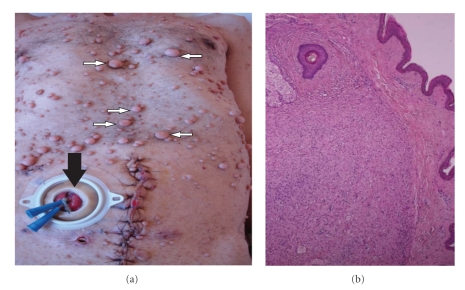
(a) Numerous cutaneous neurofibromas (small white arrows) and the performance of the ileal stoma (black arrow) located below the right line between umbilicus and the anterior superior iliac spine. (b) Microscopic view of resected cutaneous neurofibroma at the site of the performed stoma (H-E stain X 2.5).

**Figure 4:**
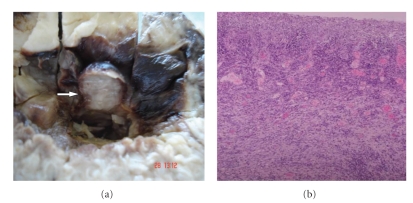
(a) Intraluminal macroscopic view of cystectomy specimen. Recognition of one out of the two polypoid lesions of urinary mucosa (white arrow). (b) Microscopic view of the ulcerated surface of the polypoid lesion (H-E stain X 2.5).

Interestingly, our patient had a recent 5-month history of suprapubic prostatectomy due to lower urinary tract symptoms, during which no bladder wall pathology was recorded. In addition, preprostatectomy imaging was reviewed and quoted as normal. Although the diagnosis of inflammatory pseudotumor could be made, the patient was consented for the uncertain biological behavior and the malignant potential of the tumor. Upon discussing definitive treatment the patient decided to undergo radical cystectomy. As a consequence, radical cystectomy followed by an ileal conduit was performed. Additionally due to lack of genetic test to confirm his inherited disease, the excised cutaneous lesions from the site of the performed stoma were sent for histopathologic evaluation (Figure 3). Macroscopic view of the cystectomy specimen revealed two polypoid lesions with smooth, elastic surface, 1 and 1,5 cm in diameter located at the bladder dome (Figure 4(a)). The ulcerated surface of the polypoid lesion was recognized microscopically (Figure 4(b)). The microscopic and immunochemistry findings confirmed the initial diagnosis after the transurethral resection of the lesion.

## 3. Discussion

Inflammatory myofibroblastic tumor or pseudotumor is a rare neoplasm believed to be benign. Among the genitourinary organs, the bladder is more commonly affected; still, this entity is extremely rare. Adult males are predominated but the disease can also affect children and elderly patients. Painless hematuria is the most common presenting symptom followed by lower urinary tract symptoms and chronic pelvic pain. Rarely does it constitute an incidental finding during radiological examination. Predisposing or exacerbating factors of genitourinary manifestation include cigarette smoking and prior surgery in the form of an endoscopic bladder surgery or an open gynecological intervention of the pelvis [6, 7]. 

IMT should be differentiated from benign lesions such as leiomyoma or solitary fibrous tumors (SFTs), and malignant lesions such as leiomyosarcoma, sarcomatoid carcinoma, or rhabdomyosarcoma. These entities share common histopathological findings. However, their clinical behavior is entirely different. To properly differentiate immunochemistry is essential. Anaplastic lymphoma kinase (ALK) which stains positive in approximately half of the IMT cases is a promising marker under investigation [1]. 

Neurofibromatosis type-1 (NF-1) is a familial autosomal dominant syndrome characterized by multiple cutaneous neurofibromas and café au lait spots, skeletal abnormalities, and nervous system manifestations. Urinary tract is rarely involved with the most common manifestation being the development of neurofibromas [8]. Two cases of non-epithelial bladder tumors, one lei*ο*myosarcoma [9] and one solitary leiomyoma [10], have been published. Alterations in the expression of NF-1 gene and of several growth factors may explain the development of mesenchymal tumors in these patients. Our patient has also been operated on and this could activate the process of tumor formation, according to the term “postoperative spindle cell nodule.” 

Bladder inflammatory myofibroblastic tumors follow a benign indolent course are treated conservatively by transurethral resection or partial cystectomy. Followup constitutes of repeated cystoscopies and biopsies to confirm complete disease remission. When IMT is clearly distinguished from aggressive lesions such as sarcomas radical surgery, radiation or chemotherapy could be considered as overtreatment [1, 7]. 

The patient was fully informed and consented to undergo radical cystectomy. The decision was based on the rarity and the unknown natural history of IMT as well as the scarce cases published on bladder tumors in Von Recklinghausen's patients. Alternative therapeutic approaches could be transurethral resection of the tumor or partial cystectomy. However the relapse rate following these treatments ranges between 10% and 30% at a mean follow-up time of approximately 30 months [6, 7]. Although radical cystectomy is an aggressive therapeutic option, minimizing the risk of grade and stage progression may optimize patient's long-term outcome.

##  Consent

Written informed consent was obtained from the patient for publication of this paper and accompanying images. A copy of the written consent is available for review by the Editor-in-Chief of this journal.

## Abbreviations

IMT: Inflammatory myofibroblastic tumor
IP: Inflammatory pseudotumor
PSCN: Postoperative spindle cell nodule
CT: Computed tomography
*α*-SMA: *α*-Smooth muscle actine
NSE: Neuron-specific enolase
SFT: Solitary fibrous tumors
ALK: Anaplastic lymphoma kinase
NF-1: Neurofibromatosis type-1.
